# Challenging Stereotypes: A Counter-Narrative of the Contraceptive Experiences of Low-Income Latinas

**DOI:** 10.1089/heq.2019.0107

**Published:** 2020-03-04

**Authors:** Diana N. Carvajal, Ruth Enid Zambrana

**Affiliations:** ^1^ Department of Family & Community Medicine, University of Maryland School of Medicine, Baltimore, Maryland.; ^2^ Department of Women's Studies, University of Maryland, College Park, Maryland.

**Keywords:** contraception, reproductive health, Latina health, immigrant health

## Abstract

**Purpose:** Reproductive autonomy is associated with educational attainment, advanced employment, and well-being. While U.S. Latinas use contraception to control their own childbearing and have reported a desire to do so, they often use it inconsistently and have the lowest rates of contraceptive use of any group. Reasons previously cited for why Latinas do not use contraception compared with non-Latino white women include lack of access, lack of knowledge, language barriers, emphasis on large families, machismo, and religiosity. These reasons are often overly simplistic and can lead to widespread generalizations about Latinas.

**Methods:** Using focus groups and semistructured interviews from November 2014 through June 2015, this study describes the family planning perspectives and experiences of 16 Latinas living in Baltimore and recruited from two federally qualified health centers. A social determinant of health framework was used to guide identification of important concepts and explain findings.

**Results:** Results demonstrated that respondents reported contraceptive agency and claimed autonomy over their bodies; described a sense of responsibility and often expressed caution about having families too large to care for; expressed educational and career aspirations; and perceived contraception as critical for the postponement of childbearing to achieve their goals.

**Conclusion:** The patient/provider encounter should include communication that recognizes all patient preferences and lived experiences to support vulnerable and/or marginalized Latinas in their desires to control their own childbearing and life choices.

## Introduction

Contraception affords control over childbearing.^1^ A person's ability to control their own reproduction is associated with educational attainment, advanced employment opportunities, and enhanced well-being of children and families.^1,2^ While U.S. Latinas use contraception and have reported a desire to do so,^2,3^ they often use it inconsistently.^3–5^ Previously noted reasons for a lack of contraceptive use or decreased consistency of use among Latinas include low knowledge about contraceptive options,^6^ language barriers,^6^ decreased access to care,^7^ a desire for large families,^3^ male partner machismo,^8,9^ and religiosity.^3^ Also important to note are experiences of perceived *discrimination* related to contraceptive services pervasive among racial/ethnic women.^10–12^ Such experiences may shape contraceptive decision-making. In addition, providers may be perceived as coercing low-income, racial/ethnic women to use certain contraceptive methods to limit childbearing, a concern previously expressed in the literature.^13^

Health care-derived and perpetuated stereotypes about low-income and poor Latinas in the United States include notions that they are uneducated, unmotivated, and/or incapable of planning ahead.^14,15^ Previous literature has observed that low-income Latinas may not have career or educational opportunities and, as a result, have no recourse except to have children at young ages.^16,17^ The notion that low-income Latinas are unable or incapable of thinking about or having life trajectories beyond those that include early childbearing can typecast them as possessing few assets. Other approaches to studying Latinas challenge previous scholarship about negative stereotypes often attributed to low-income Latinas.^18–21^ Author Lorena Garcia tells the stories of young, Chicago Latinas who confronted existing racist stereotypes and social/cultural constructs that marginalized them on the basis of socioeconomic status—to instead assert their sexual and reproductive agency by practicing safe sex.^22^ Garcia argues that Latinas possess sexual agency; we assert that they also possess *aspirational wealth*^23^ when it comes to childbearing and family planning. Aspirational capital is the ability to maintain desires and ambitions despite unfavorable current circumstances and significant obstacles.^23^ Common stereotypes about Latinas—especially those related to family planning and childbearing—may subvert their agency and aspirational capital.

In this study, we describe the family planning perspectives and experiences of young Latinas residing in Baltimore, MD, guided by three questions: (1) in what ways do participants exercise agency in their use of contraception; (2) what are the perceptions of participants with respect to childbearing and family size; and (3) what are Latinas' aspirations beyond childbearing? The goal was to identify how the lived experiences of young Latinas impact family planning and childbearing decisions within the context of their life plans.

## Methods

When considering how existing stereotypes about low-income Latinas might impact family planning, we chose to use a social determinant of health (SDH) framework^24^ not only to guide identification of important concepts but also to explain our findings. The SDH framework acknowledges that health inequities result from social inequities, often as a consequence of racism, discrimination, and implicit bias.^24^ Moreover, inequity and disadvantage often start before birth and accumulate throughout life, and are associated with long-term health outcomes.^24^ An SDH framework recognizes that effective health care delivery requires participation and decision-making by communities empowered by individuals within communities.^24^ Reducing inequity requires healthy individuals and communities whose potential is maximized by assuring them control over their lives.^24^

### Study design, study setting, sample, and recruitment

Focus groups and semistructured interviews occurred in Baltimore; participants were recruited from two federally qualified health centers (FQHCs) that provide care to a majority low-income population of patients. Approximately 75% of the patient population of the FQHCs is insured through public assistance, and 35% of the total patient population self-reports as Latino. Purposive sampling^25^ was used to recruit participants who self-identified as Latina, were ages 15 to 24 years, and were not pregnant or intending to become pregnant at the time of the interview. Potential participants were approached in the waiting room of the facilities by a trained, bilingual research assistant after checking in for a visit.

A total of 30 potential participants were approached; 27 agreed to be screened for eligibility, and 25 were found eligible to participate and were subsequently consented. Participants provided written, signed informed consent (≥18 years old) or written, signed informed parental consent and assent (<18 years old). Of the consented Latinas, 16 participated (9 were lost to follow-up as the time of focus group/interview was scheduled for a separate occasion). Eight young women agreed to participate in focus groups, while the other 8 participated in individual interviews. At the outset of this study, researchers planned to conduct focus groups only; however, after 3–4 months of recruitment, participant scheduling difficulties, transportation issues, and family/work responsibilities posed significant barriers to their ability to commit to focus group meeting times. In addition, some focus group participants seemed uncomfortable discussing sensitive topics in the presence of other participants and were often overshadowed by more vocal participants. To address these practical and research issues,^26^ researchers conducted additional individual interviews. This study was approved by the University of Maryland Baltimore Institutional Review Board.

While the sample size may seem small, this study was meant to be exploratory and responses yielded consistent themes that reached repetitive thematic responses early on (i.e., *data saturation*^27^). Prior research suggests that 6–12 interviews per homogeneous group often yield sufficient data to achieve saturation.^28^

### Focus group and interview procedures

Focus groups and interviews were conducted between November 2014 and June 2015. Groups were held separately for younger participants (those 15–19 years old) and for older participants (20–24 years old). There were three mini focus groups (*n*=3; *n*=3; *n*=2) and eight individual interviews. Each participant attended only one group or one interview. Groups/interviews were held in private spaces. Each participant received a $20 gift card for her time.

Focus groups lasted ∼60 min; individual semistructured interviews ranged from 30 to 45 min. Discussions were facilitated in both English and Spanish based on participant language preference by a trained, bilingual, self-identified Latina moderator. Questions aimed to identify participants' perspectives about childbearing and contraception use.

### Analysis

Discussions were audio-recorded and professionally transcribed verbatim. Spanish transcripts were translated and then transcribed to English. A native Spanish-speaking researcher compared all original Spanish transcripts with the corresponding translated versions to confirm accuracy. Transcripts were analyzed by listening to each recording, and then reading the Spanish transcription followed by the English transcription. Two researchers independently coded each of the English transcripts. Memos were written and themes were identified through a line-by-line analysis of the data. Investigators discussed their findings with attention to major concepts of the SDH framework. These major concepts were used as a guide to organize data, generate initial codes, develop a coding scheme, and identify connections among constructs.

## Results

### Description of sample

Sixteen adolescent and young adult women, ages 15 to 24 years, were either part of three focus groups (*n*=3; *n*=3; *n*=2) or had individual interviews (*n*=8). Ten participants were 15–19 years old; 6 participants were 20–24 years old. Participants were born in the following countries: Mexico (*n*=3), Honduras (*n*=6), El Salvador (*n*=3), Colombia (*n*=1), Dominican Republic (*n*=1), Brazil (*n*=1), and the United States (*n*=1). The average length of time in the United States was 5 years. Only the participant from Brazil chose to speak English during her interview. Fifteen participants chose to speak Spanish during the focus group/interview.

### Themes

Three central themes emerged. Each theme is presented with illustrative quotations. To provide richer descriptions of the lived experiences of respondents, complete, additional representative quotations are displayed in Table 1.

**Table 1.** Quotations Illustrating Three Major Themes

Question	SDH concept	Themes	Quote examples
(1) What are Latinas' aspirations beyond childbearing?	All children and young people are entitled to maximize their full potentials.	Aspirational capital	An 18-year old from Honduras [translated from Spanish]: “I'm with my partner and I have contraceptives and I'm thinking about my future, I want to study, I want to keep my job, I want to have all this before I have a family, so the advantage of all this that comes with contraceptives is that you can think and prepare, plan.” A 16-year old from Mexico [translated from Spanish]: “They [young women] have goals to reach and if they get pregnant, that's going to prevent them from reaching their goals, and for a girl that's hard…” A 20-year old from Brazil: “…we're trying to figure our lives out… we're like allowing ourselves to find real jobs, find career paths, get our education, and as much as we—we usually, like, tend to like babies and little kids …having a baby in our lives seems to put everything on a hold and not allow ourselves to figure out who we are… figure out what we would like to do, what career we'd like to have for the rest of our lives… —yeah. I think that's why we tend to use it [contraception].” A 19-year old from El Salvador [translated from Spanish]: “… in these times, I think it is good to prevent [pregnancy]… like I said, there are many young girls, at an early age, they get pregnant, and they lose school, they are left with nothing.”
(2) In what ways do participants exercise agency in their use of contraception?		Agency	An 18-year old from Honduras [translated from Spanish]: “… you need to …be in control and everything so you don't get pregnant so fast.” A 17-year old from Dominican Republic [translated from Spanish]: “I know the church disagrees with them [contraceptives]… I go to church, but I don't disagree with [them] because it helps people who are not ready. Because if they are active sexually speaking, they might get pregnant… That's why I don't disagree with contraceptives.” A 24-year old from Honduras [translated from Spanish]: “…I really feel like it's [using contraception] a personal decision. Because if it helps you, then okay, take it. It's going to be beneficial for your health.” An 18-year old from Honduras [translated from Spanish]: … “her [a friend] partner did not want her to plan. So, she made an appointment without him knowing.” An 18-year old from Honduras [translated from Spanish]: “…there are parents that are very religious and so, um, they don't allow their daughters to use contraceptives because they think, oh if they're using this they have sex and they don't agree with their sons or daughter being sexually active at such an early age, so they tell them no, you can't use contraceptives, thinking that if they tell them no they're going to stop having sex but that's a mistake because they're taking away the opportunity for them to protect themselves.” A 16-year old from Mexico [translated from Spanish]: “Umm, my mom, she agrees very much that I use them [contraceptives] because she knows that at my age, many girls and boys… want to experiment… and she prefers that I take care of myself, because tomorrow she doesn't want me to dedicate myself more to my son or my daughter than to my school, that in life I have a job, a family and something to give to that child, so she agrees pretty much that I use them.” A 24-year old from Colombia [translated from Spanish]: “Evangelical Christians have more the idea of ‘Wait for your husband or remain virgin until your marriage.’ But we need to be realistic in this world… If one has the role of a parent, it is better to talk to your kids about contraception.”
(3) What are the perceptions of participants with respect to family size?		Sense of Responsibility	A 24-year old from Honduras [translated from Spanish]: “…if you're not emotionally prepared to have a child, then it's going to have a toll on the child's life… if you want to be sexually active but you're not prepared to have a child, then I think that's a smart decision [using contraception] so you won't be putting the child's emotional life, like, in a danger type of deal… because that way, that cannot lead the child into like negative effects.” A 16-year old from Mexico [translated from Spanish]: “Sometimes the advantage [of using contraception] is that it gives you time to prepare for life… it [contraception] lets you prepare in life for when you want to have a baby, and the disadvantages is that perhaps yes you'll gain weight, your hair falls out or sometimes it gives you like a headache. But I prefer to gain weight, that my hair falls out, or that I get headaches to losing tomorrow the opportunity of studying and become someone in life that can give my son or my daughter a house, a place to live.” A 20-year old from Brazil: “Umm, because people [children] do need your time. People do need your affection and everything that you have… Uh, it [pregnancy] can happen at the end, towards the end when you're like almost there—getting your PhD—yeah. But not while you're trying to get your education, I think. Because I think it would be unfair to your family—because you would always be giving yourself to school, always having a reason why you can't make it to the family events type of thing. So, I think of it like that. It's like not being fair to the people around you.” An 18-year old from Honduras [translated from Spanish]: “… for me I think it [contraception] is beneficial because sometimes there are too many children in one family and they can't adjust and all that… Because then when, I mean, if you don't plan and everything, you'll get pregnant quickly, you'll have very young children and all that and if you don't make the proper adjustments, that's when you start having family problems. Yeah, I am in favor [of using contraceptives].” A 24-year old from Honduras [translated from Spanish]: “…if you're simply not ready to have a child, then I think it's [using contraception] a really smart decision. You're not holding yourself back, you're not, um, putting another person's—the child's life at like, risk—yeah.”

SDH, social determinant of health.

#### Theme 1: contraception agency

The first theme demonstrated that participants had *contraceptive agency*. Most participants claimed autonomy over their bodies and refuted the notion that other individuals or entities could control them. They seemed to exercise agency in their use of contraception, countering stereotypes that overbearing, overly religious families and machismo influence their contraceptive decisions. Most asserted that decisions to use contraception were theirs and not of others such as family members, male partners, or religion. While supportive family members and partners were viewed in a positive light, the opinions of those who opposed contraceptive use did not affect participants' decisions to use it. Using contraception was viewed as a personal decision. Decisions about contraception were often made without consulting family members or partners and irrespective of religious doctrine.

“… you need to …be in control and everything so you don't get pregnant so fast.” (18-year-old, Honduras; translated from Spanish)

“I know that the church disagrees with them [contraceptives]… I go to church, but I don't disagree with contraceptives because it is a help for those people who are not ready. Because if they are active sexually speaking, they might get pregnant…” (17-year-old, Dominican Republic; translated from Spanish)

“…I really feel like it's [using contraception] a personal decision… (18-year-old, Honduras; translated from Spanish)

“… her [a friend] partner did not want her to plan. So, she made an appointment without him knowing.” (18-year-old, Honduras; translated from Spanish)

These data show that participants were seeking to assert control over their reproductive lives; the data provide additional support for the SDH framework, which advocates for the empowerment of young people and adults to have control over their lives.^24^ Accordingly, findings support the notion that the health care system should work to support young women in their decisions to assert control over their family planning.

#### Theme 2: family planning responsibility

A second theme demonstrated that participants exhibited a sense of *responsibility*. During discussions about family size, a majority of participants expressed caution about having large families for which they may not ultimately be able to provide. They invoked the notion of responsibility in childbearing as important. They feared not being able to care for their children and some participants asserted that having children for whom they could not provide would be unfair [to the children]. A majority of respondents described potential burdens of parental responsibility and asserted that while having large families is not a necessity, taking responsibility for family *is*.

“…if you're not emotionally prepared to have a child, then it's going to have a toll on the child's life… if you want to be sexually active but you're not prepared to have a child, then I think that's a smart decision [using contraception] so you won't be putting the child's emotional life, like, in a danger type of deal” (24-year-old, Honduras; translated from Spanish)

“Sometimes the advantage [of using contraception] is that it gives you time to prepare for life… it [contraception] lets you prepare in life for when you want to have a baby, and the disadvantage is that perhaps yes you'll gain weight, your hair falls out or sometimes it gives you like a headache. But I prefer to gain weight, that my hair falls out, or that I get headaches to losing tomorrow the opportunity of studying and become someone in life that can give my son or my daughter a house, a place to live.” (16-year-old, Mexico; translated from Spanish)

#### Theme 3: educational and career aspirations

The third theme affirmed respondents' *aspirational capital.* Participants almost uniformly (all but one) reported that contraception is necessary because of aspirations to achieve educational and career goals, affirming their abilities to maintain ambitions despite significant life obstacles. Most participants voiced the importance of attaining educational and career goals before childbearing.

“I'm with my partner and I have contraceptives and I'm thinking about my future. I want to study. I want to keep my job. I want to have all this before I have a family……” (18-year-old, Honduras; translated from Spanish)

“They [young women] have goals to reach and if they get pregnant, that's going to prevent them from reaching their goals, and for a girl that's hard…” (16-year-old, Mexico; translated from Spanish)

“…we're trying to figure our lives out… we're like allowing ourselves to find real jobs, find career paths, get our education, and as much as we—we usually, like, tend to like babies and little kids …having a baby in our lives seems to put everything on a hold and not allow ourselves to figure out who we are… figure out what we would like to do, what career we'd like to have for the rest of our lives…” (20-year-old, Brazil)

These respondents illustrated a desire and capacity to plan for their futures. Findings suggest that health care providers should consider asking about young Latinas' aspirations when counseling about family planning—thereby supporting them to choose the methods they deem best. In line with the SDH framework,^24^ the above quotes exemplify an underlying set of values that all youth should have the opportunity to maximize their capabilities. Moreover, for health care providers, translating these findings into effective patient-centered counseling can promote equitable care.

These data provide a counter-narrative to the stereotypes sometimes seen in the literature on Latinas' reproductive health and decision-making. In their agency, they take control of their reproductive health decisions in an effort to achieve certain goals they believe will improve their social circumstances and that of their future children. These findings provide rich insight into Latina youth perceptions of the responsibility associated with childbearing. Our data demonstrate that prior literature may be based on incomplete data and missing narratives.

## Discussion

A significant body of work on women's reproductive decision-making has overlooked the role of agency in decisions—particularly for low-income racial/ethnic women. Some studies have identified culture and language as the main drivers of life decisions,^15,29^ while others have attributed differences in life choices to socioeconomic status.^16,30,31^ Our findings contribute to a thread of research that focuses on agency. Data demonstrate that low-income Latinas are indeed capable of engaging in thoughtful choices about contraception and childbearing with respect to their life trajectories. Their choices are often guided by educational and career aspirations despite their marginalized status as low-income Latinas. Aspirational capital is observed to be foundational in driving their sense of reproductive agency.

Findings also offer insights about the factors that shape participants' claim for control of their lives. Reproductive decisions are often independent of (or influenced little by) religion, family pressures, male partners, and community beliefs. Important barriers that may derail family planning are provider discrimination and poor-quality communication. A solid trajectory of studies has exposed the role of discriminatory practices and inequity in family planning access and counseling services.^10–12,18,32,33^ Racial/ethnic-based stereotypes are perpetuated and can affect the quality of service perceived by patients. Such stereotypes can undermine patient agency and their trust and communication with health care providers—both factors closely associated with patient-centered care.^34^ In alignment with Marmot's SDH framework, it is important to recognize that the identification of health inequities is strongly associated with social status and racism.^24,35,36^

For centuries, poor racial/ethnic women have been blamed for not assuming responsibility for their family decisions with disregard for the role of institutional structures and policies in depriving women of agency.^32,33^ These discriminatory practices included denial of contraceptive access, forced sterilization of low-income, racial/ethnic women,^32,33,37^ and stereotypic attitudes.^38^ Our data encourage alternative pathways of investigation that center the paradigm on the lived experiences of low-income Latinas as being autonomous drivers of their reproductive health.

Study limitations include that data on household income or education level were not available. However, since participants were recruited from an FQHC that serves predominately low-income minority patients, the site serves as a proxy for low household income. As more than half of the participants were teenagers (19 years old and younger), a significant percent of the sample had less than a high school education. Other limitations included a lack of information about the socioeconomic status of participants' families, their sources of social support, and available community resources. In addition, given the nature of the discussion, there was a risk of social acceptability bias. Most importantly, participants are not representative of all low-income Latinas whether immigrant or native-born, and these results are not generalizable.

## Conclusion

Implications of this research are important for informing health policy and translating evidence into clinical practice as stipulated by Marmot's *Social Determinants of Health* framework.^24^ Social and economic disadvantages accumulate throughout life—which can impact health across generations.^24^ Therefore, it makes sense for health care providers to bolster positive health determinants such as aspiration and agency to strengthen the opportunity of low-income, marginalized women to control their own childbearing. Moreover, counseling women in ways that align with their personal preferences and aspirations can ultimately impact health inequities and is key for achieving social justice. Providers should avoid imposing their own values during contraceptive counseling and instead should focus on understanding unique perspectives on childbearing, forging trust, and providing care that is responsive to personal needs.

Within the context of social justice, a framework of *reproductive justice* is also paramount in the delivery of equitable care. Ross defines reproductive justice as “the complete physical, mental, spiritual, political, economic, and social well-being of women and girls.”^32^ Historically and in contemporary times, low-income African American and Latino women perceive race- and ethnicity-based discrimination when accessing reproductive health services.^10,11,39^ Yet, the central issues facing marginalized populations are not just about unplanned pregnancy, but also the intersection of socioeconomic and racial/ethnic injustices that allow for some to have reproductive autonomy while others cannot.^40^ Generalized assumptions among family planning providers regarding the contraceptive preferences of low-income immigrant Latinas counter the advancement of a reproductive justice framework. The perpetuation of stereotypes about Latinas propagates discrimination and inequity in family planning care and hinders the translation of evidence into effective and responsive clinical practice.

Ultimately, clinicians cannot provide quality care while continuing to perpetuate stereotypes about particular groups. Our data suggest a shift in how we approach family planning counseling for low-income Latinas. Personal preferences, lived experiences, and/or goals should inform counseling practices. Our data align with the SDH framework asserting that we must promote and support healthy, sustainable communities to eliminate health inequities.^24^ Supporting the SDH framework, we argue that the promotion and sustainability of health equity are central to the achievement of reproductive and social justice.

## Abbreviations Used

FQHCs: federally qualified health centers
SDH: social determinant of health
